# The Role of NF-kB in the Downregulation of Organic Cation Transporter 2 Expression and Renal Cation Secretion in Kidney Disease

**DOI:** 10.3389/fmed.2021.800421

**Published:** 2022-01-04

**Authors:** Chao Han, Juan Zheng, Fengyi Wang, Qingyang Lu, Qingfa Chen, Ankang Hu, Michele Visentin, Gerd A. Kullak-Ublick, Zhibo Gai, Lei Chu

**Affiliations:** ^1^Department of Nephrology, Tengzhou Central People's Hospital, Zaozhuang, China; ^2^Department of Joint Laboratory for Translational Medicine Research, Liaocheng University/Liaocheng People's Hospital, Liaocheng, China; ^3^Department of Urology, Tengzhou Central People's Hospital, Zaozhuang, China; ^4^Department of Pathology, Liaocheng People's Hospital, Liaocheng, China; ^5^Institute for Tissue Engineering and Regenerative Medicine, Liaocheng University/Liaocheng People's Hospital, Liaocheng, China; ^6^Laboratory Animal Center, Xuzhou Medical University, Xuzhou, China; ^7^Department of Clinical Pharmacology and Toxicology, University Hospital Zurich, University of Zurich, Zurich, Switzerland; ^8^Innovation Research Institute of Traditional Chinese Medicine, Shandong University of Traditional Chinese Medicine, Jinan, China; ^9^Department of Urology, The Affiliated Tengzhou Hospital of Xuzhou Medical University, Zaozhuang, China; ^10^Department of Urology, Affiliated Tengzhou Hospital of Jining Medical University, Zaozhuang, China

**Keywords:** chronic kidney disease, inflammation, NF-kB, organic cation transporter, TNF-α, tubular secretion

## Abstract

Organic cation transporter 2 (OCT2), encoded by the *SLC22A2* gene, is the main cation transporter on the basolateral membrane of proximal tubular cells. OCT2 facilitates the entry step of the vectorial transport of most cations from the peritubular space into the urine. OCT2 downregulation in kidney disease models is apparent, yet not clear from a mechanistic vantage point. The aim of this study was to explore the role of inflammation, a common thread in kidney disease, and NF-kB in OCT2 modulation and tubular secretion. Among the OCTs, OCT2 was found consistently downregulated in the kidney of rats with chronic kidney disease (CKD) or acute kidney injury (AKI) and in patients diagnosed with CKD, and it was associated with the upregulation of TNFα renal expression. Exposure to TNFα reduced the expression and function of OCT2 in primary renal proximal tubule epithelial cells (RPTEC). Silencing or pharmacological inhibition of NF-kB rescued the expression of OCT2 in the presence of TNFα, indicating that OCT2 repression was NF-kB-dependent. *In silico* prediction coupled to gene reporter assay demonstrated the presence of at least one functional NF-kB *cis*-element upstream the transcription starting site of the *SLC22A2* gene. Acute inflammation triggered by lipopolysaccharide injection induced TNFα expression and the downregulation of OCT2 in rat kidney. The inflammation did reduce the active secretion of the cation Rhodamine 123, with no impairment of the glomerular filtration. In conclusion, the NF-kB pathway plays a major role in the transcriptional regulation of OCT2 and, in turn, in the overall renal secretory capacity.

## Introduction

Solutes are excreted from the body via the kidneys through three concerted processes: glomerular filtration, tubular secretion and reabsorption (1–3). For decades, glomerular filtration was considered the predominant process in renal excretion, yet from an evolutionary standpoint, tubular secretion appears fundamental, with glomerular filtration evolving only afterwards (4). Only later, tubular secretion was demonstrated to be critical for the clearance of organic ions and even involved in the elimination of salt and water (5, 6). Tubular secretion occurs, for the most part, at the proximal tubule level, and, as demonstrated in stop-flow and micropuncture seminal studies in mammalian, fish, and avian kidneys, it seems particularly relevant for positively charged solutes including endogenous substances, xenobiotics and drugs, the latter accounting for close to 40% of approved compounds with marketing authorization by regulatory agencies (7). The first step of cation tubular secretion, the translocation of the substrate from the interstitial space into the proximal tubular cell, is often rate-limiting and it is primarily mediated by the organic cation transporters 1, 2 and 3 (OCT1-3), Na^+^-independent, polyspecific transporters expressed on the basolateral membrane of proximal tubular cells (8). Extensive pharmacogenetic evidence indicates that among the OCTs, OCT2 is the primary route of renal elimination of several drugs to the point that guidance of drug regulatory agencies demands that each molecule in development be tested *in vitro* for inhibition of OCT2 transport activity in order to predict potential drug-drug interactions (9, 10).

It has been recently shown that, among patients with end-stage kidney disease, protein-bound substances, which are prominently eliminated by active tubular secretion, accumulate in substantially higher concentrations compared to free solutes that are mainly filtered (11). Serum creatinine, which is the staple marker of kidney function, is a substrate of OCT2, which may be accountable for up to 30% of the overall creatinine clearance (12). Likewise, Trimethlyamine-N-oxide (TMAO) level, an OCT2 substrate (13), was found markedly higher in the plasma of patients with chronic kidney disease receiving dialysis than in the plasma of healthy individuals. Moreover, renal transplantation resulted in substantial reductions in TMAO plasma concentration (14), suggesting that OCT2 activity might be somewhat reduced in patients with CKD. Indeed, the renal OCT2 expression seems to be highly affected by the underlying pathological context. Different animal models of kidney damage were characterized by a lower expression of Oct1 and Oct2 and a reduced clearance of OCT substrates such as cimetidine, famotidine and tetraethylammonium (TEA) as compared with the control animals (3, 15–17). Conversely, mice fed a high-fat diet with reduced kidney function were characterized by a higher expression of Octs in comparison with lean animals (18). The modulation of OCT expression in kidney disease is likely of pleiotropic nature and may involve alteration in various protein kinase activity and activation of the ischemia/reperfusion-inducible protein (IRIP) (19–22). The recently published results of the Chronic Renal Insufficiency Cohort (CRIC) study on 3,939 participants revealed that biomarkers of inflammation inversely correlated with the measures of kidney function and positively with albuminuria (23). In the present study, we investigated the role of local inflammatory state, a common thread in the evolution of diseases with distinct etiopathogenesis different such as CKD and AKI (24), and its main transcriptional mediator NF-kB, in the regulation of the expression of OCTs and the pathological and pharmacological ramifications thereof.

## Materials and Methods

### Patient Characteristics

Diagnosis of CKD was made according to the residual renal function assessed per serum creatinine level and the Kidney Disease Improving Global Outcomes (KDIGO) classification (Table 1). Renal biopsies were obtained from five patients with chronic kidney disease (CKD). The patient was placed in the prone position; the puncture point was marked at the level of the lower kidney. Under local anesthesia, the needle was inserted into the renal capsule under ultrasound guidance, and the renal cortex tissue was ejected from the puncture needle. Pathologically normal samples were isolated from the tissue surrounding the tumor removed from ten patients who underwent partial nephrectomy at the Liaocheng University/Liaocheng People's Hospital between December 2019 and December 2020.

**Table 1:** Clinical and demographic data of patients.

	**Normal (***n*** = 10)**	**CKD (***n*** = 5)**	***P*** **-value**
Age (years)	53.9 ± 4.40	46.8 ± 6.83	0.038
Gender (M/F)	6/4	1/4	0.282
Serum Creatinine (μmol/L)	59.1 ± 4.24	132.8 ± 5.85	0.0007
eGFR (mL/min/1.73 m^2^)	102 ± 8.93	43.8 ± 4.55	0.0007
**Category (%)**
G1	10 (100)	0 (0)	
G2	0 (0)	0 (0)	
G3a	0 (0)	3 (60)	
G3b	0 (0)	2 (40)	
G4	0 (0)	0 (0)	
G5	0 (0)	0 (0)	

*Data are expressed as the mean ± SD. CKD, chronic kidney disease; eGFR, estimated glomerular filtration rate; M, male; F, female; sCr, serum creatinine. eGFR was calculated with the formula: = 141 x min (sCr/κ, 1)^α^ x max (sCr /κ, 1)^-1.209^ x 0.993^age^ x 1.018 [if female] x1.159 [if Black]. κ = 0.7 (females) or 0.9 (males). α = −0.329 (females) or−0.411 (males). Min, indicates the minimum of sCr/κ or 1; Max, indicates the maximum of sCr/κ or 1*.

### Animal Housing and Generation of the Experimental Models

Only male rats were used in this study because the expression level of OCT2 in the kidneys of male rats is higher than that in female rat kidneys (25, 26). Male Sprague-Dawley rats (200–220g) from Vital River Laboratories (Beijing, China) were placed in the animal facility with a 12:12-h day and night cycle and ad libitum food (AIN93 M, Keao Xieli feed co. LTD, Beijing China) and water. Chronic kidney disease (CKD) was induced by a two-stage procedure of 5/6 nephrectomy with lateral incision to expose the retroperitoneal kidney, as previously described (27). All rats were sacrificed under anesthesia 4 weeks after the second stage of surgery, kidneys were excised and halved by longitudinal incision to expose the cortex, which was isolated for further analyses. Acute kidney injury (AKI) was induced by unilateral with contralateral nephrectomy, as previously described (28). Briefly, the right-sided nephrectomy or sham procedure was performed, then the left renal artery was carefully dissected and occluded for 30 min using a non-traumatic microvascular clamp. All rats were humanely sacrificed by CO_2_ inhalation, 24 h after the surgery. Kidneys were harvested and halved by longitudinal incision to expose the cortex, which was isolated for further analyses. For each disease model, sham operations were carried out by performing a lateral incision without disturbing the internal organs, under the same anesthesia conditions. Inflammation was induced by daily intraperitoneal injection of 5mg/kg body weight endotoxin lipopolysaccharide (L2880, LPS from Escherichia coli, Sigma-Aldrich, St. Louis, MO) for 3 days. Control animals received the same dose of detoxified LPS (dLPS), uncoupled from the biologically active component lipid A, essential for the activation of inducible nitric oxide synthases (iNOS) (29). LPS and dLPS were dissolved in sterile Hank's Balanced Salt Sodium (HBSS, Gibco, UK) supplemented with 10 mM HEPES (Gibco, UK), and adjusted to pH 7.4 with NaOH. Twelve hours after the last injection, three animals per group were euthanized by CO_2_ inhalation, the kidneys harvested for mRNA, and protein expression studies, two animals per group were subjected to the Rhodamine 123 excretion assay and then sacrificed by CO_2_ inhalation.

### Cell Culture

Human RPTEC (Human Renal Proximal Tubule Epithelial Cells) cell line was obtained from Lonza (Basel, Switzerland). Cells were grown in renal epithelial cell growth medium bullet kit (CC-3190, Lonza, Basel, Switzerland) supplemented with 10% fetal bovine serum (FBS), 100 units/ml penicillin, and 100 μg/ml streptomycin. For differentiation, the percentage of FBS was reduced from 10 to 2%.

### Rhodamine 123 Excretion Assay

The urinary excretion of Rhodamine 123 was monitored as previously reported (30). Rats were hydrated by subcutaneous injection of 1 ml 0.9% NaCl 1 h before the initiation of the perfusion. The perfusion was performed under anesthesia with i.p. pentobarbital (60 mg/kg, Southland Pharmaceutical, Guangzhou, China). Furosemide (10 mg/kg, Southland Pharmaceutical, Guangzhou, China) was injected i.p. to prevent the deterioration of the distal nephron. Heparin (125 IU/100 g, Qianhong Bio-pharma, Changzhou, China) was injected into the spleen to reduce the risk of blood clot formation. The renal artery of the right kidney was cannulated via the mesenteric artery without interruption of the blood flow, and a cannula was inserted into the ureter. The excised kidney was connected to a recirculation perfusion system, which was oxygenated with a 95% O_2_/5% CO_2_ mixture and placed in a fluid bath with a constant temperature of 37.5°C. The perfusion fluid consisted of: 112.0 mM NaCl, 5.2 mM KCl, 2.0 mM CaCl_2_, 1.0 mM MgCl_2_, 25 mM NaHCO_3_, 0.84 mM Na_2_HPO_4_, 0.28 mM KH_2_PO_4_, 5.0 mM D-glucose, 4.0 mM urea, 25.0 g/l pluronic F108, 0.33 mM glutathione, 0.083 mM Myo-inositol, 0.50 mM cysteine, 2.3 mM glycine, 2.0 mM Na-pyruvate, 1.22 mM Na-acetate, 0.21 mM Na-propionate, 1.0 mM inosine, 5.0 mM alanine, 0.11 mM Na-glutaminate, 2.0 mM L-glutamine, 0.01 mM ascorbic acid, 1.0 mM Na-lactate, 1.0 mg/l choline chloride, 4 IU/l insulin, 2.0 μg/l aldosterone, 10 μg/l anti-diuretic hormone, 20.0 μg/l andangiotensin II. To this solution, 8.5% Travasol (1B6624, Baxter, Shanghai, China) was added to a final concentration of 0.1%. After perfusion for 30 min to equilibrate the kidney, the perfusion fluid was supplemented with a solution containing Rhodamine 123 (R8004, Sigma-Aldrich, St. Louis, MO) to a final concentration of 525 nM. Urine samples were collected every 10 min. Perfusate samples were drawn at the midpoint of each urine collection interval. For the determination of the glomerular filtration rate (GFR), tetramethylrhodamine isothiocyanate-dextran (T1037, TRITC-Dextran, 11.4 μM, 4400 mol wt, Sigma-Aldrich, St. Louis, MO, USA) was added to the perfusion fluid 10 min after the stabilization period. The concentrations of Rhodamine 123 and TRITC-dextran in urine samples were determined directly after the experiment by fluorescence spectrophotometry using a Perkin Elmer LS50B (Perkin Elmer, Beaconsfield, Buckinghamshire, UK).

### Isolation of RNA From Kidney Tissue and Quantification of Transcript Levels

Total RNA was isolated using Trizol (ThermoFisher scientific, Waltham, Massachusetts, USA) according to the manufacturer's instruction. After DNAse (Promega, Madison, WI, USA) treatment, two μg of total RNA was reverse transcribed using oligo-dT primers and Superscript II (ThermoFisher scientific, Waltham, Massachusetts, USA). Complementary DNA (cDNA) was used for real-time polymerase chain reaction analysis with TaqMan master mix and primers (Applied Biosystems, CA, USA). Transcript levels, determined in two independent complementary DNA preparations, were calculated and expressed relative to the housekeeping gene b-actin. The TaqMan probes used in this study are: human OCT1, Hs00427550_m1; OCT2, Hs00533907_m1; OCT3, Hs01009568_m1; TNFa, Hs00174128_m1; iNOS: Hs01075527_m1; SGLT1: Hs01573793_m1; SGLT2: Hs00894642_m1; b-ACTIN, Hs99999903_m1; rat Oct1, Rn00562250_m1; Oct2, Rn00580893_m1; Oct3, Rn00570264; Tnfa, Rn99999017_m1, Mdr1: Rn01639253_m1; Mate1: Rn01460731_m1; b-actin, Rn00667869_m1.

### Immunostaining

Human and rat kidney tissues were fixed in 4% paraformaldehyde for 1 h and then kept at 4 degree until paraffin embedding. Kidney paraffin sections (4-μm-thick) were hydrated, microwaved for 8–15 min in 10 mM sodium citrate (pH 6.0) for antigen retrieval, and then probed with a rabbit antibody against OCT2 (1:100, LS-C357581, LSBio, Seattle, WA, RRID:AB_2891045), OCT3 (1:100, LS-C406026, LSBio, Seattle, WA, RRID:AB_2891046) or TNFα (1:100, ab6671, Abcam, Cambridge, United Kingdom, RRID:AB_305641). Immunolabeled sections were then incubated with goat anti-rabbit second antibody conjugated to horseradish peroxidase and treated with the EnVision^+^ diaminobenzidine kit (DAB, Dako, Glostrup, Denmark) using standard protocols. An unbiased observer assessed the staining score of 5-6 random high-power fields according to area and intensity of the positive staining. The cross-sectional areas with positive staining were determined based on the percentage of positive cells: <5% (0); 5–25% (1); 25–50% (2); 50–75% (3); >75% (4). Staining intensity grades as follows: no staining (0); light yellow (1); yellow brown (2); dark brown (3).

### Western Blotting

Cells and tissues were homogenized using RIPA lysis buffer (89900, ThermoFisher scientific, Waltham, MA, USA). Twenty μg of protein lysates were resolved by SDS-PAGE and blotted onto polyvinylidene difluoride membranes (Millipore, Burlingtob, MA, USA). The membranes were incubated overnight at 4 °C with the primary antibodies followed by incubation with the respective secondary antibodies. Membranes were developed using the ECL Plus detection system (32209, ThermoFisher scientific, Waltham, MA, USA). Primary antibodies used were anti-OCT2 (1:1000, LS-C357581, LSBio, Seattle, WA, RRID:AB_2891045) and Na^+^-K^+^ ATPase (1:1000, sc-16041, Santa Cruz, Dallas, TX, United States, RRID:AB_647701). As secondary antibodies, goat anti-rabbit IgG (HRP) (ab6721, Cambridge, United Kingdom, RRID:AB_955447) or donkey anti-goat IgG (HRP) (ab97110, Cambridge, United Kingdom, RRID:AB_10679463) were used.

### Construction of Human OCT2 Promoter Vectors

The part of the sequence of the human OCT2 promoter containing the predicted NF-kB binding site was cloned from a commercially available human genomic library (G1471, Promega, Fitchburg, WI, USA) using the forward primer 5'-cccgacggctcttgttgttggttg-3' and the reverse primer 5'-gcgcgaaggtagccgagagcaga-3'. A 689-bp amplicon corresponding to the−586 / +103 fragment flanking the transcription starting site of the *SLC22A2* gene was isolated and blunt ligated into the pTargeT vector (A1410, Promega, Madison, WI, USA). The insert was then subcloned in pMCS-Luc vector (16146, ThermoFisher scientific, Waltham, MA, USA) by digestion with NheI and Smal restriction enzymes. Mutants were generated using AvaII restriction enzyme to generate the Δ327 construct or the FauI restriction enzyme to generate the Δ363 vector. The TTTCAAAA-mutant was generated by QuikChange Site-Directed Mutagenesis Kit (A13282, Thermofisher, La Jolla, CA, USA).

### Luciferase Assay

RPTEC cells were seeded into 24-well plates at a density of 1.5 x 10^5^ cells/well. Cells were transfected using Lipofectamine 2000 (ThermoFisher scientific, Waltham, Massachusetts, USA), with 600 ng of DNA and 30 ng of a control vector for transfection efficiency, using the constitutive expression pCMV-Red Firefly Luc Vector (16156, ThermoFisher scientific, Waltham, MA, USA). After transfection, cells were exposed to 10 ng TNFα (< 0.1 endotoxin units per μg of protein, 210-TA, R&D Systems, Minneapolis, MN, USA) or the vehicle control for 24 h and then luciferase activity was determined using a Gaussia-firefly luciferase dual assay kit (16181, ThermoFisher scientific, Waltham, MA, USA) and a LB940 luminometer (Berthold, Germany).

### NF-KB p65 Transcription Activity Assay

NF-kB p65 DNA binding activity from nuclear extracts were determined by using ELISA-based colorimetric kit from Abcam (ab133112, Cambridge, UK) as previously described (31). Nuclear extracts from cells were incubated overnight with a double stranded DNA sequence containing the NF-kB response element prior ELISA.

### Statistics

Data are expressed as mean ± S.D. Statistical significance of differences between groups was assessed by Student's *t*-test or one-way ANOVA test. GraphPad (RRID: SCR_002798) was used to perform statistical analyses.

### Study Approval

Animal protocols, and experiments abided by the laws of animal protection and were approved by the local institutional animal committee of Xuzhou Medical University, China (license number 20210102W001). The handling of human samples followed the Declaration of Helsinki guidelines regarding ethical principles for medical research involving human subjects. Written informed consent was provided by each patient participated in the research. The study protocol was approved by the Scientific Ethical Committee of Liaocheng University/Liaocheng People's Hospital, China (license number 2020022).

## Results

### Organic Cation Transporter Expression Level in CKD and AKI

**Figure 1:**
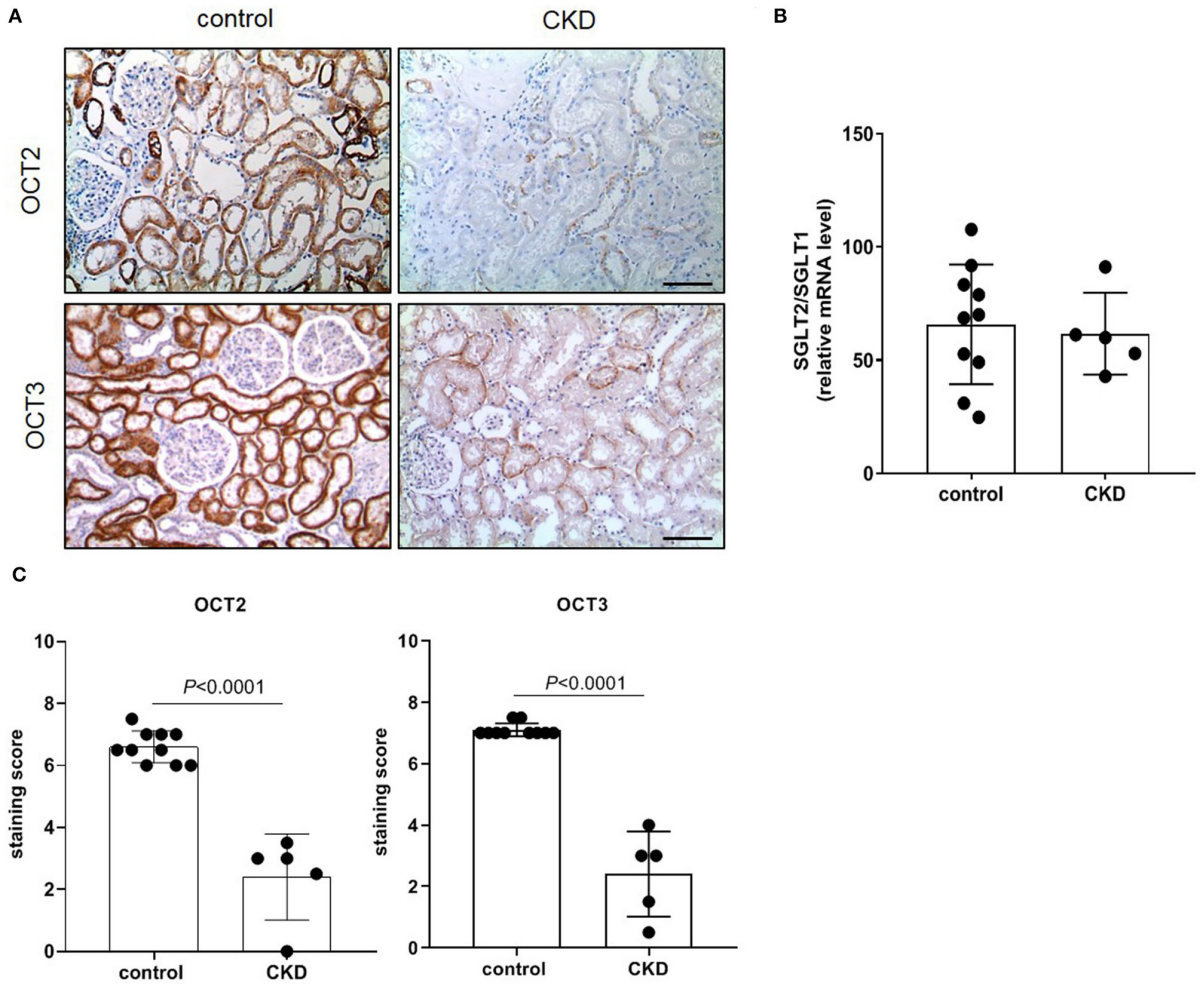
Immunostaining of human OCT2 and OCT3. Kidney biopsies were obtained from patients diagnosed with chronic kidney disease (CKD). Normal tissue available from partial nephrectomy in patients diagnosed with renal cancer were used as control. Representative images of immunostaining for OCT2 and OCT3 **(A)**. Scale bar = 50 μm. The ratio of SGLT2/SGLT1 mRNA level was used as control of the site of the biopsy. Data were normalized for the expression level of β-actin, used as housekeeping gene, and expressed as mean ± SD **(B)**. Relative quantification of the OCT2 and OCT3 staining per high-power field **(C)**. All comparisons were performed by unpaired Student *t*-test. In scatter plot representation, each dot represents one individual sample.

**Figure 2:**
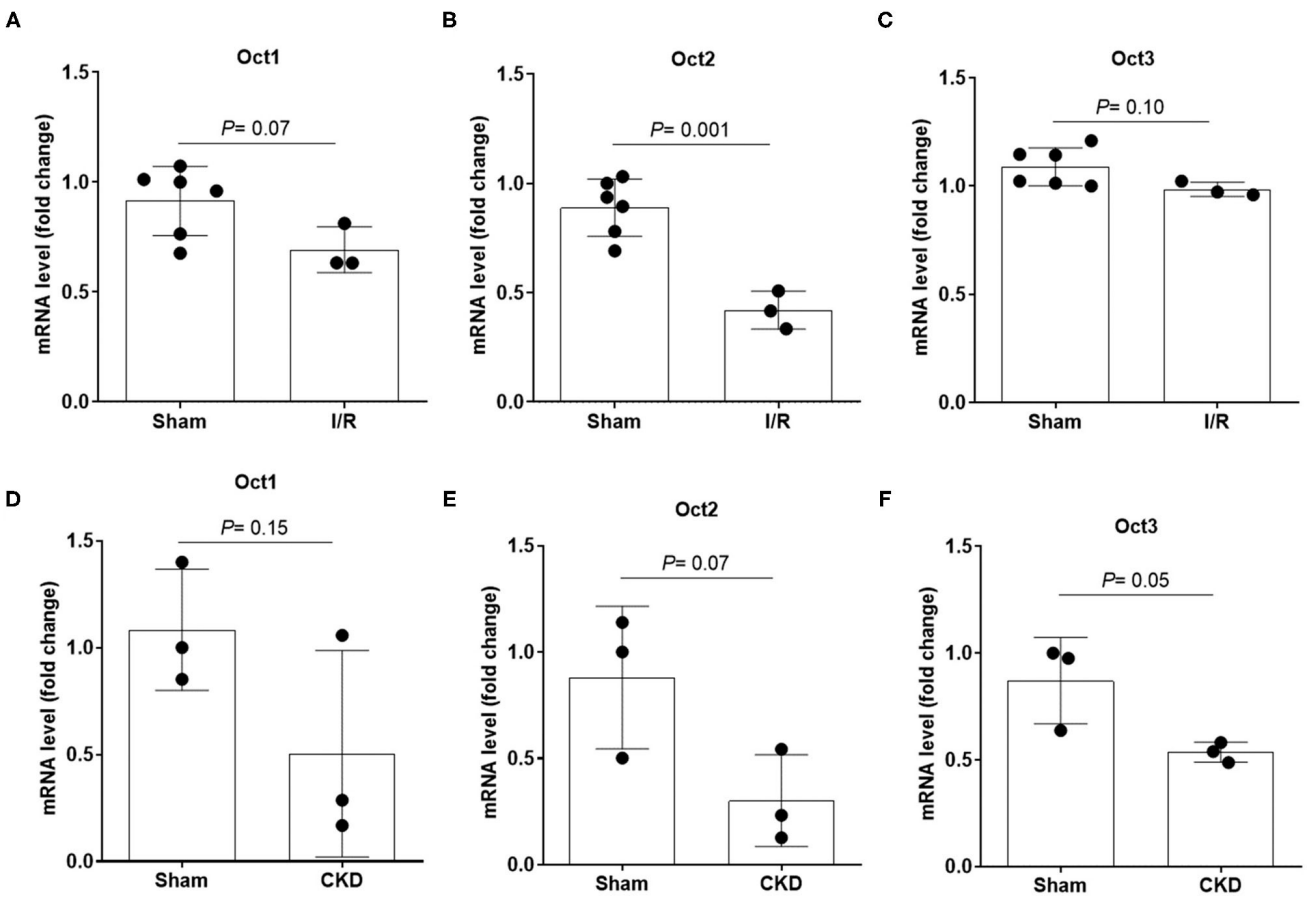
mRNA expression level of Oct1, Oct2 and Oct3 in rat kidney. Relative quantification of mRNA levels of Oct1, Oct2 and Oct3 in kidney of rats that underwent 5/6 nephrectomy to induce CKD **(A–C)** or unilateral with contralateral nephrectomy to induce AKI **(D–F)**. Data were normalized for the expression level of β-actin, used as housekeeping gene, and expressed as mean ± SD relative to sham values. All comparisons were performed by unpaired Student *t*-test. In scatter plot representation, each dot represents one individual sample.

**Figure 3:**
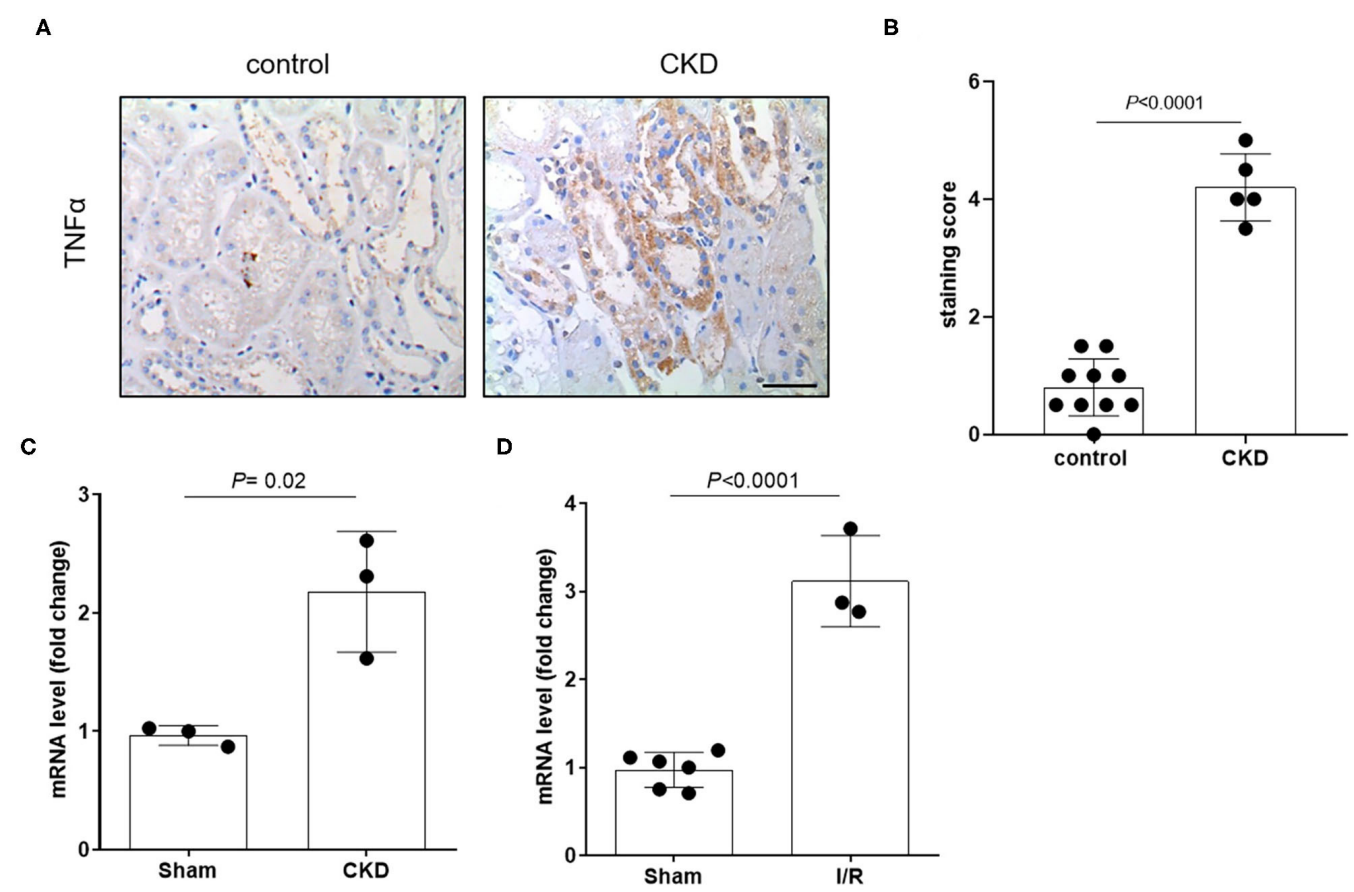
Expression level of TNFα in patients and in animals. Representative image **(A)** and relative quantification **(B)** of TNFα staining in kidney biopsies obtained from patients diagnosed with chronic kidney disease (CKD). Normal tissue available from a partial nephrectomy in patients diagnosed with renal cancer were stained as control. Scale bar = 100 μm. Relative quantification of mRNA levels of Tnfα in kidney of rats that underwent 5/6 nephrectomy to induce CKD **(C)** or unilateral with contralateral nephrectomy to induce AKI **(D)**. Data were normalized for the expression level of β-actin and expressed as mean ± SD relative to sham values. All comparisons were performed by unpaired Student *t*-test. In scatter plot representation, each dot represents one individual sample.

The protein expression level of OCT2 and OCT3, the two main polyspecific organic cation transporters expressed in human proximal tubular cells, was evaluated in biopsies from patients diagnosed with CKD and compared to that in pathologically normal renal tissue collected from patients with normal kidney function who underwent surgery for suspicion of renal cancer. Figure 1 shows that OCT2 and OCT3 localized on the basolateral membrane of proximal tubular cells and that the protein expression level of both transporters was markedly lower in the kidney of CKD patients than that in the kidney of patients with normal renal function. The subcortical origin of the bioptic tissues was determined by assessing the mRNA level of the sodium/glucose cotransporter 1 (SGLT1), whose expression is confined to segment 3 of the cortex, and the sodium/glucose cotransporter 2 (SGLT2), expressed in segments 1 and 2, where OCT2 and OCT3 primarily localize. In Figure 1B, it can be seen that the mRNA level of SGLT2 exceeded that of SGLT1 by approximately 50-fold in the control as well as in the CKD group, supporting the outer cortex origin of the samples. Analogously, in line with previous reports (3, 15–17), Oct2 mRNA expression level was markedly reduced in CKD and I/R-induced rats (Figure 2). Conversely, the renal expression level of TNFα, localized in the cytoplasm, was significantly increased in patients diagnosed with CKD (Figures 3A,B) as well as in rats with CKD or AKI (Figures 3C,D).

### Effect of Recombinant TNFα on the OCT Expression in RPTEC Cells

**Figure 4:**
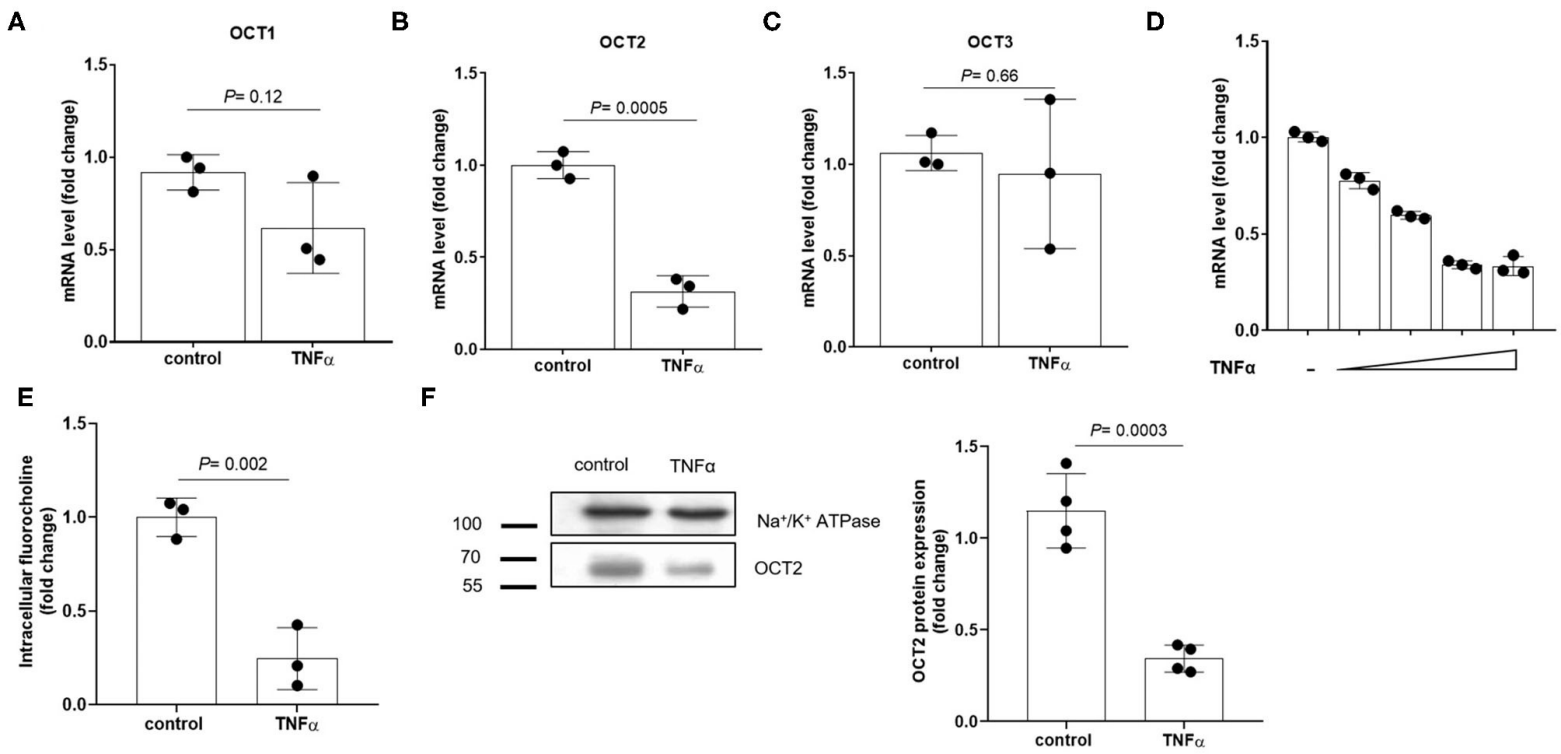
Expression of OCTs in RPTEC cells exposed to TNFα. Relative quantification of mRNA levels of OCT1 **(A)**, OCT2 **(B)** and OCT3 **(C)** in RPTEC cells exposed for 24 h to recombinant TNFα at the extracellular concentration of 10 ng/ml. mRNA expression level of OCT2 in RPTEC cells exposed for 24 h to increasing extracellular concentrations of recombinant TNFα (2, 5, 10, 20 ng/ml) **(D)**. All mRNA data were normalized for the expression level of β-actin and expressed as mean ± SD relative to the control values. Uptake of fluorocholine, an OCT substrate, in RPTEC cells exposed for 72 h to recombinant TNFα at the extracellular concentration of 10 ng/ml **(E)**. Representative western blotting and quantification relative to the expression of the Na^+^/K^+^ ATPase of OCT2 from the total lysate of RPTEC cells exposed for 72 h to recombinant TNFα at the extracellular concentration of 10 ng/ml **(F)**. In scatter plot representation, each dot represents one biological replicate.

**Figure 5:**
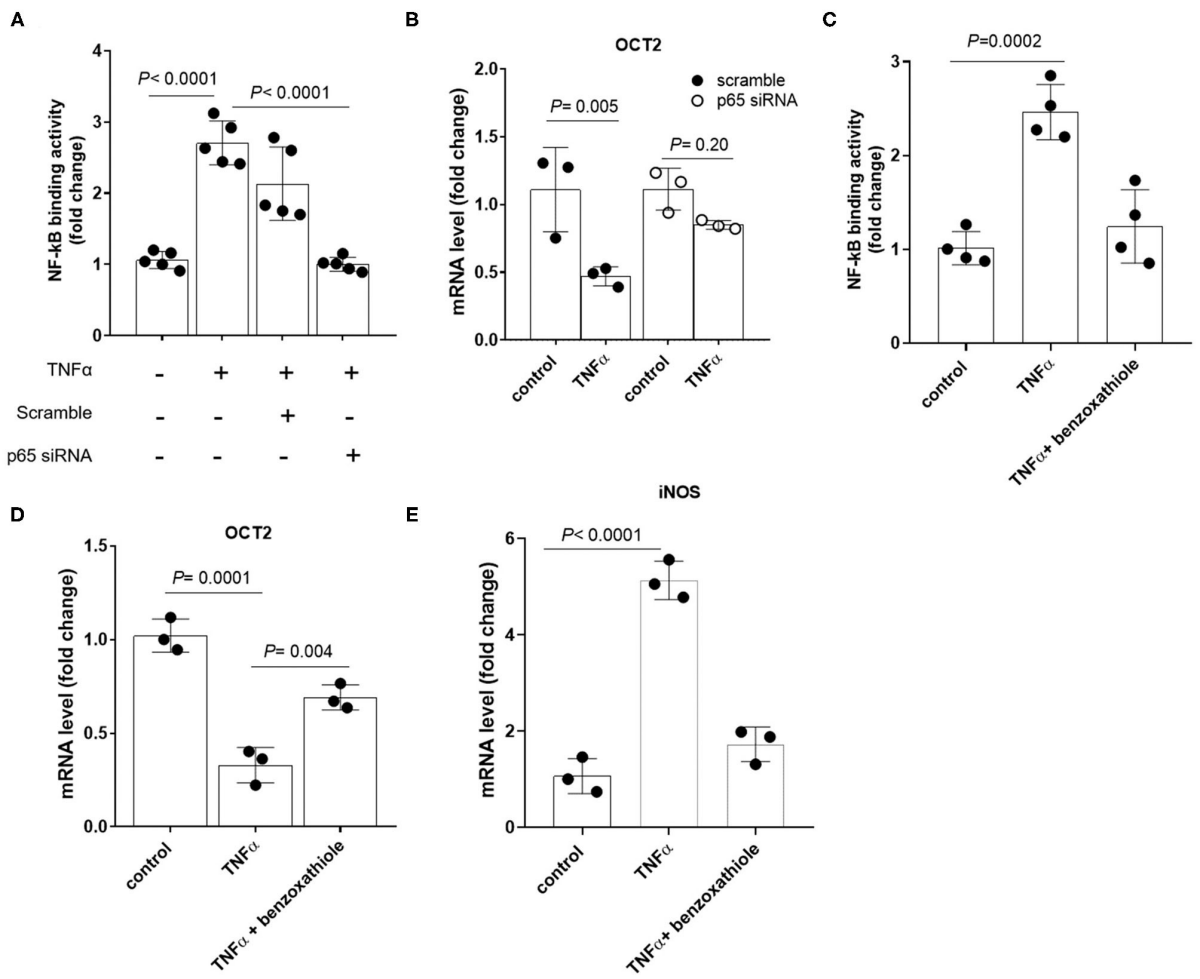
Expression of OCT2 in RPTEC cells exposed to TNFα upon disruption of NF-kB activation. NF-kB p65 binding activity **(A)** and OCT2 mRNA level **(B)** in RPTEC cells transiently transfected with scramble or p65 siRNA followed by 24h-exposure to recombinant TNFα at the extracellular concentration of 10 ng/ml. NF-kB p65 binding activity **(C)**, OCT2 **(D)** and iNOS **(E)** mRNA level in RPTEC cells exposed to recombinant TNFα at the extracellular concentration of 10 ng/ml in the presence or absence of the NF-kB inhibitor Benzoxathiole at the extracellular concentration of 10 μM. All mRNA data were normalized for the expression level of β-actin and expressed as mean ± SD relative to the control values. In scatter plot representation, each dot represents one individual experiment.

To investigate the existence of a causal link between TNFα induction and the repression of OCTs, RPTEC cells were exposed to recombinant TNFα at the extracellular concentration of 10 ng/ml. In Figure 4 it can be seen that OCT2 mRNA level (B) was markedly decreased in the presence of TNFα, whereas those of OCT1 (A) and OCT3 (C) did not significantly change. The TNFα-induced downregulation of OCT2 was dose-dependent with maximal response at 10 ng/ml (Figure 4D). To investigate the impact of TNFα on OCT2 function, the protein expression level and the transport of fluorocholine, primarily an OCT2 substrate (32, 33), was assessed upon 72 h-exposure to recombinant TNFα at the extracellular concentration of 10 ng/ml. It can be seen that fluorocholine uptake (Figure 4E) as well as OCT2 protein level (Figure 4F) were markedly reduced by the incubation with TNFα. Thus far, the inhibitory effect of pro-inflammatory cytokines on OCT2 expression was considered a post-translational modulation, however, the present data suggest that TNFα also repressed the transcription of the *SLC22A2* gene, encoding for OCT2 (34). Being NF-kB the primary genuine transcription factor activated by TNFα, its role in TNFα-induced OCT2 downregulation was investigated. The incubation with recombinant TNFα enhanced NF-kB binding activity in RPTEC cells (Fig. 5A). To assess whether the TNFα-induced OCT2 downregulation was NF-kB dependent, the NF-kB transcriptional activity was disrupted by silencing p65, the NF-kB subunit responsible for initiating transcription subunit (Figure 5B). The repressing effect of recombinant TNFα on OCT2 gene expression was abolished in RPTEC cells transiently transfected with a siRNA against p65, indicating that the downregulation of OCT2 expression upon exposure to TNFα required a functional NF-kB. Similarly, treatment of RPTEC cells with benzoxathiole, a NF-kB inhibitor (Figure 5C), partially restored the OCT2 gene expression in the presence of recombinant TNFα (Figure 5D). Notably, benzoxathiole also almost fully abolished the induction of the expression of the inducible nitric oxide synthase (iNOS), which was previously shown to reduce OCT2 protein expression (Figure 5E) (34).

### Identification of NF-KB Binding Site in OCT2 Promoter

**Figure 6:**
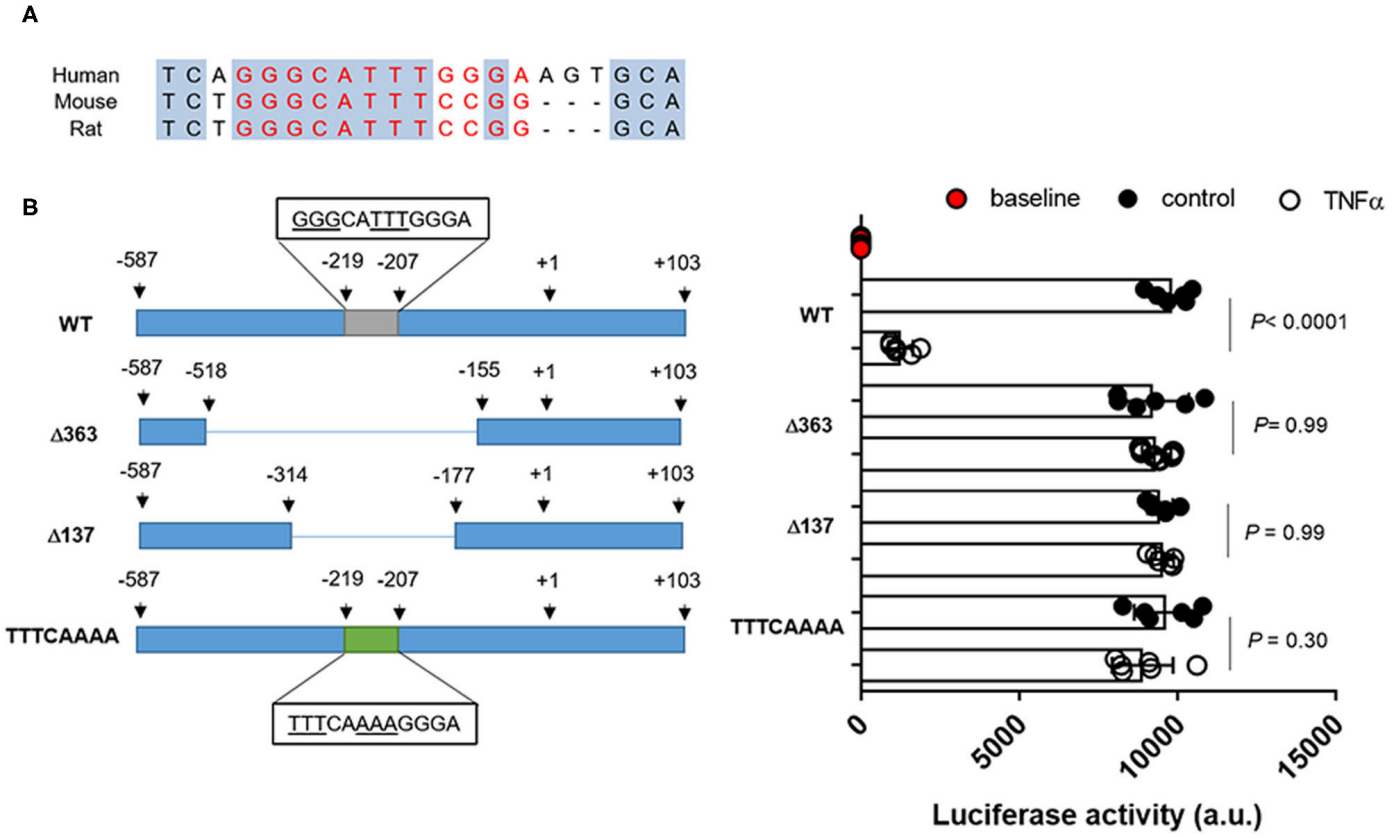
OCT2 promoter activity in the presence of TNFα. Putative NF-kB binding site in human and rodents **(A)** Promoter activity in RPTEC cells transfected with the pMCS-Red Firefly Luc containing the wild-type or the mutated sequence of the OCT2 promoter and exposed to 10 ng/ml TNFα or the vehicle control for 24 h. The fluorescent signal emitted by empty vector-transfected cells not exposed to TNFα was considered the baseline activity. Data were normalized for the pCMV-Red Firefly Luc Vector control activity and expressed as the mean ± S.D. from three independent experiments performed in duplicate. All comparisons were performed by one-way ANOVA followed by Tukey's *post-hoc* test was employed **(B)**.

In rat Oct2 promoter region, a putative NF-kB binding site in position−143 to−129 was previously predicted (26). Based on sequence alignments, a similar sequence was found in position−219 to−207 of the promoter region of the human OCT2 (Figure 6A). As shown in Figure 6B, the activity of the OCT2 promoter region containing the putative NF-kB binding site was significantly suppressed in the presence of recombinant TNFα. Such inhibition was abolished by deleting or mutating the putative NF-kB binding site (Figure 6B).

### Effect of LPS Injection on Organic Cation Secretion

**Figure 7:**
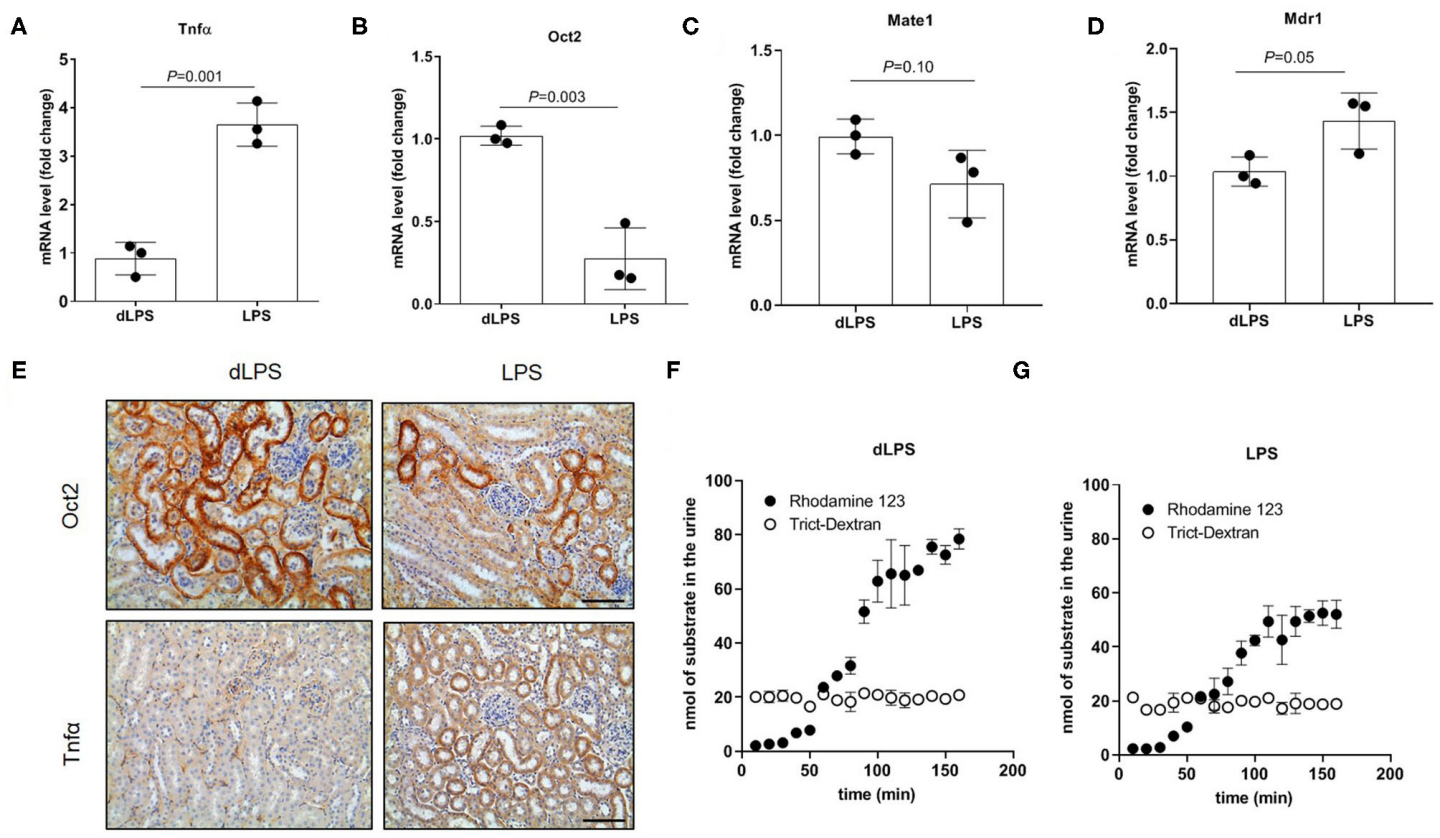
Tubular secretion in rats treated with lipopolysaccharide (LPS). Relative quantification of mRNA **(A–D)** and representative immunostaining of protein expression level **(E)** in kidneys of rats injected with LPS. Animal controls were injected with the same amount of detoxified LPS (dLPS). Scale bar = 50 μm. mRNA data were normalized for the expression level of β-actin and expressed as mean ± SD relative to the dLPS values. Urinary excretion over time of Rhodamine 123 and Trict-Dextran in rats (*n* = 2) **(F,G)**. All comparisons were performed by unpaired Student *t*-test. In scatter plot representation, each dot represents one individual sample.

To assess the direct effect of inflammation on OCT expression and organic cation tubular secretion, rats were treated with LPS to induce acute inflammation. In Figure 7 it can be seen that the mRNA (Figure 7B) and the protein (Figure 7E) of Oct2 was significantly reduced by the treatment with LPS, conversely, the mRNA (Figure 7A) and protein (Figure 7E) level of Tnfα was markedly higher in the kidney of rats exposed to LPS. The kidney mRNA level of Mate1 and Mdr1 (P-glycoprotein), two apical transporters often coupled to Oct2 in the vectorial transport of cations, was not significantly different between LPS and control animals (Figures 7C,D). To investigate whether the LPS injection affected the renal handling of organic cations, the fluorescent cationic compound Rhodamine 123 was used as a tracer. Rhodamine 123 has been previously shown to be substrate of OCT2 (35), which mediates Rhodamine 123 tubular secretion (34, 36). The renal handling of Rhodamine 123 was determined in the kidney 12 h after the last injection of LPS. Figure 7F shows that the accumulation of Rhodamine 123 over time exceeded that of tetramethyl Rhodamine isothiocyanate (TRITC)-conjugated dextran (TRITC-Dextran), a fluorescent high molecular mass dextran which is not reabsorbed and is considered a practical read-out of glomerular filtration (37). It can be seen that, at steady-state level, the renal clearance of Rhodamine 123 was close to 4 times that of TRICT-Dextran, indicating that Rhodamine 123 is actively secreted by the tubule. The steady-state renal clearance of Rhodamine 123 in the rats treated with LPS was roughly 60% of that in rats treated with dLPS (Figures 7F,G).

## Discussion

The expression of oct2 and cation active tubular secretion is often altered in various animal models of kidney disease. In rats, oct2 expression level and the clearance of the quaternary ammonium cation Tetraethylammonium (TEA), a prototypical OCT substrate, decreased after induction of acute kidney injury by ischemia-reperfusion procedure (17). Similarly, 5/6 nephrectomized rats developing chronic renal failure had lower expression of oct2 and slower tubular secretion of cimetidine, another OCT substrate (3). Oxonate-induced hyperuricemic rats (15), as well as diabetic rats (16) displayed a downregulation of oct2. Conversely, our groups showed that obese individuals and mice fed a high-fat diet with chronic kidney disease had a higher expression of renal oct2 than lean ones (18). The present work demonstrates that acute inflammation in the frame of normal renal function is sufficient to repress the expression of oct2, resulting in a slower active tubular secretion of the fluorescent cation Rhodamine 123. OCT2 mediates the transport of the substrate from the peritubular blood into the proximal tubular cells, and works in tandem with the multidrug extrusion transporter 1 (MATE1) and with efflux pump such as P-gp, both located on the brush-border membrane of the proximal tubular cells (38, 39). While MATE1 has been previously shown to be downregulated by TNFα (40), no significant change in MATE1 mRNA level was observed when RPTEC cells were exposed to TNFα (not shown), nor in the kidney of rats treated with LPS (Figure 7C). Similarly, we did not observe LPS-mediated induction of the mRNA level of P-gp as previously reported (30). Taken together, our data indicate that OCT2 is rate limiting in Rhodamine 123 vectorial transport across the proximal tubule (12, 41–43).

The effect of endotoxemia on Oct-mediated tubular secretion has been previously put forth by Heemskerk and colleagues, who demonstrated that aminoguanidine, an inhibitor of the inducible nitric oxide synthase (iNOS), rescued the tubular secretion of Rhodamine 123 and the protein expression of oct1 and oct2, but not that of their mRNA, in the kidney of Wistar Hannover rats injected with LPS (34). The induction of iNOS expression upon binding and nuclear translocation of NF-kB (Figure 5E) (44), boosts the conversion of l-arginine into nitric oxide, a gas that mediates multiple biochemical processes, including the activation of the guanylate cyclase, thereby the increase synthesis of cyclic guanosine monophosphate (cGMP) a signaling molecule that was previously shown to be involved in the inhibition of the activity of rat oct1 and human OCT2 *in vitro* (45–47). Here, we show that the effect of TNFα on OCT2 expression was abolished upon silencing of p65, the NF-kB subunit responsible for initiating transcription and upon pharmacological inhibition of NF-kB with benzoxathiole. This suggests that TNFα-induced downregulation of OCT2 occurs exclusively via NF-kB and it is the result of the iNOS-independent *SLC22A2* gene transcription repression and the iNOS-dependent reduced protein stability, with only the latter rescued by the treatment with aminoguanidine (34).

In the present work, we focused on elucidating the renal TNFα-OCTs axis, yet the repressive effect of pro-inflammatory cytokines has been reported for various other transporters including bile acid transporters, organic anion transporters (OATs), organic anion transporting polypeptides (OATPs) and OCT1 (48). Therefore, the reduction in tubular secretion induced by inflammation may not be restricted to organic cation, but rather encompasses a wider spectrum of solutes, including various putative uremic toxins observed in patients with CKD (49). Genetic, life-style and environmental factors can directly trigger the disease, which slowly creates a highly oxidative uremic milieu that fosters the inflammatory cycle and worsens the residual renal function (50). Notably, inhibitors of the renin–angiotensin–aldosterone system, such as angiotensin-converting enzyme inhibitors (ACEIs) and angiotensin II receptor blockers (ARBs), which are widely prescribed in the management of CKD, seems also to reduce the production of pro-inflammatory products by, at least in part, inhibiting the NF-kB pathway (24). In a similar vein, the inhibition of prostaglandin synthesis by non-steroidal anti-inflammatory drugs (NSAIDs) increased the tubular solute reabsorption in healthy volunteers (51). In line with this, administration of IL-1 receptor antagonist in hemodialysis patients significantly reduced systemic inflammation markers and the urinary loss of albumin (52). In line with these clinical observations, our previous study demonstrated that genetically TNFα-deficient mice fed a high-fat diet were resistant to renal inflammation and oxidative stress in comparison with animals fed a normal diet (53). An efficient control of the local inflammatory status may sustain the tubular secretion of protein bound uremic toxins escaping the hemodialysis, which, despite major technological advances, remains a major limitation of today's renal replacement therapy (49).

## New and Noteworthy

Despite the advance of the technology, hemodialysis and hemofiltration are not able to compensate for the reduced active tubular secretion of protein-bound substances suffered by patients with kidney dysfunction. Our experimental results coupled to the observed association between OCT expression and pro-inflammatory cytokines in the kidneys of patients diagnosed with chronic kidney disease; provide the rationale for implementing a long-term anti-inflammatory therapy alongside the standard renal replacement therapy.

## Data Availability Statement

The raw data supporting the conclusions of this article will be made available by the authors, without undue reservation.

## Ethics Statement

The studies involving human participants were reviewed and approved by the Scientific Ethical Committee of Liaocheng University/Liaocheng People's Hospital, China (license number 2020022). The patients/participants provided their written informed consent to participate in this study. The animal study was reviewed and approved by the Local Institutional Animal Committee of Xuzhou Medical University, China (license number 20210102W001).

## Author Contributions

CH, JZ, and LC: conception and design. CH, JZ, FW, QL, QC, AH, MV, and ZG: methodology and data acquisition. CH, JZ, GK-U, and LC: data interpretation. CH and LC: writing of the initial draft. JZ, FW, QL, QC, AH, MV, GK-U, and ZG: revision and editing. All authors have read and approved the final manuscript.

## Funding

This work was supported by the Teacher's Research of Jining Medical University to LC (JY2017FS034), and by the Natural Science Foundation of Shandong Province to QC (ZR2020KH026).

## Conflict of Interest

The authors declare that the research was conducted in the absence of any commercial or financial relationships that could be construed as a potential conflict of interest.

## Publisher's Note

All claims expressed in this article are solely those of the authors and do not necessarily represent those of their affiliated organizations, or those of the publisher, the editors and the reviewers. Any product that may be evaluated in this article, or claim that may be made by its manufacturer, is not guaranteed or endorsed by the publisher.
